# Association between cognitive function and ambient particulate matters in middle-aged and elderly Chinese adults: Evidence from the China Health and Retirement Longitudinal Study (CHARLS)

**DOI:** 10.1016/j.scitotenv.2022.154297

**Published:** 2022-07-01

**Authors:** Yifan Yao, Kai Wang, Hao Xiang

**Affiliations:** Department of Global Health, School of Public Health, Wuhan University, 115# Donghu Road, Wuhan 430071, China; Global Health Institute, School of Public Health, Wuhan University, 115# Donghu Road, Wuhan 430071, China

**Keywords:** Particulate matter, Cognitive function, Middle-aged and elder adults, Longitudinal study

## Abstract

Increasing studies have discussed how ambient air pollution affects cognitive function, however, the results are inconsistent, and such studies are limited in developing countries. To fill the gap, in this study, we aimed to explore the effect of ambient particulate matters (PM_1_, PM_2.5_, PM_10_) on cognitive function of middle-aged and elderly Chinese adults. A total of 7928 participants older than 45 were included from CHARLS collected in 2011, 2013, and 2015. Cognitive function was evaluated with two dimensions, the first one was episodic memory and the second dimension was mental status. The total score of cognitive function was the sum of above two dimensions (0–31 points). Participants' exposure to ambient particulate matters was estimated by using a satellite-based spatiotemporal model. Linear mixed models were applied to analyze the impact of PM_1_, PM_2.5_, and PM_10_ on cognition function. Further interaction analyses were applied to examine the potential effect modifications on the association. After adjusting for confounding factors, we found an IQR increase in all three ambient particulate matters was significantly associated with a decrease in cognitive function score, with the greatest effect in the 90-day exposure window for PM_1_ (β = −0.227, 95%CI: −0.376, −0.078) and PM_2.5_ (β = −0.220, 95%CI: −0.341, −0.099). For ambient PM_10_, the most significant exposure window was 60-day (β = −0.158, 95%CI: −0.274, −0.042). Interaction analyses showed that the PM-cognitive function association could be modified by gender, region, alcohol consumption, smoking, education level, chronic diseases, and depressive symptoms. In conclusion, exposure to ambient particulate matter for a certain period would significantly decrease cognitive function among middle-aged and elderly Chinese. Furthermore, individuals who were female, or lived in the midland of China were more susceptible to the adverse effect of particulate matters.

## Highlights

• Exposure to ambient particulate matters (PM) would decrease cognitive function.
• Living in midland of China may be more prone to cognitive decline after PM exposure.
• Females were more susceptible to the adverse effects of PM on cognitive decline.

## Graphical abstract

**Figure:**
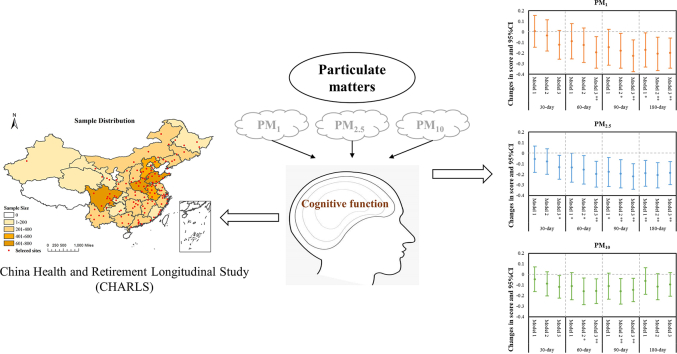

## 1 Introduction

Mild cognitive impairment (MCI) is currently considered a syndrome of cognitive decline beyond what would be expected from an individual's age and education level (Gauthier et al., 2006). MCI is an unstable transitional state between normal aging and dementia, which contains a variety of possible outcomes, ranging from stabilization and improvement to further conversion to dementia (Panza et al., 2007; Petersen, 2004). It is of great significance for the early diagnosis and prediction of dementia. Previous study showed that cognitive function declines with ages (Seshadri and Wolf, 2007). And the world's population is aging rapidly, the aging population in both developed and developing countries is expected to continue to grow (Beard et al., 2016; Gerland et al., 2014). Rapid aging increases the burden of cognitive impairment (Hebert et al., 2013; Weuve et al., 2014), age-related cognitive impairment has become a global public health problem. Therefore, identifying the risk factors of cognitive impairment can provide the scientific basis for disease prevention and control.

Many risk factors lead to cognitive impairment, such as genetic predisposition (Schram et al., 2007) and cardiovascular disease (Baumgart et al., 2015). However, a few studies have recently focused on the association between air pollution and cognitive impairment (Griffiths and Mudway, 2018; Paul et al., 2019; Schikowski and Altuğ, 2020), and some studies demonstrated the effects of air pollution on cognitive function from a mechanistic perspective (Allen et al., 2017; Nicholson et al., 2022). A study in Mexico from the National Survey of Health and Nutrition showed that exposure to PM_2.5_ positively associated with cognitive decline in old adults (Salinas-Rodríguez et al., 2018). A cohort study showed that exposures to long-term PM_2.5_ and NO_2_ were associated with decreased cognitive function among National Social Health and Aging Project (NSHAP) participants (Tallon et al., 2017), the results showed that an IQR increase in 7-year PM_2.5_ exposures was associated with a 0.25 (95% CI: −0.43, −0.06) point decrease in the Chicago Cognitive Function Measure (CCFM) scores, while an IQR increase in 7-year NO_2_ exposure with a −0.27 (95% CI: −0.48, −0.07) point decrease in CCFM scores. Most of above studies have been conducted in developed and low-pollution countries (Ailshire and Clarke, 2015; Ailshire and Crimmins, 2014; Kulick et al., 2020b; Power et al., 2011); similar studies are rarely carried out in China and other developing countries.

Due to the low level of economic development and the relative lag in environmental protection, the aging problem and environmental problems in developing countries are relatively severe. And prior studies found an increased risk for cognitive impairment in middle-aged (45 ≤ age < 60) (Gottesman et al., 2017) and elderly (age ≥ 60) (Ailshire and Clarke, 2015; Ailshire and Crimmins, 2014; Kulick et al., 2020a; Paul et al., 2019). Therefore, it is necessary to explore the association between air pollution and cognitive function among middle-aged and elderly in developing country. This type of research is of far-reaching social significance for solving health problems such as environment and aging.

In this longitudinal study, we aimed to investigate the relationship between ambient particulate matters (PM_1_, PM_2.5_, PM_10_) and cognitive function of middle-aged and elderly Chinese adults. All the participants were followed-up from China Health and Retirement Longitudinal Study Wave 1 to Wave 3.

## 2 Materials and methods

### 2.1 Data collection

Our study is a longitudinal study based on data from China Health and Retirement Longitudinal Study (CHARLS). The CHARLS is a national, longitudinal survey among residents aged over 45 in China from 2008 to 2018, which includes assessment of demographic backgrounds, health status and functioning, social and economic status, and retirement information. Supported by the multistage probability sampling method, the CHARLS research team surveyed middle-aged and elderly community residents from 28 provinces in China (Zhao et al., 2014b). The national baseline survey (Wave 1) including 17,708 participants was conducted in 2011, Wave 2 and Wave3 were followed in 2013 and 2015. A total of 7928 participants were selected in this cohort study after excluding those aged below 45 years, lost to follow up and missing data on cognitive function. The full process of participants' selection is depicted in Fig. S1.

### 2.2 Assessment of cognitive function

Cognitive function was measured from two dimensions (Lei et al., 2014; Luo et al., 2019), the first dimension is episodic memory divided into immediate word recall (0–10 point) and delayed word recall (0–10 point). The second dimension was mental status based on questions of the Telephone Interview of Cognitive Status (TICS) battery, which established to understand the integrity or mental state of the individual. Mental status was measured from orientation, visuo-construction, and attention. Orientation (0–5 points) was evaluated by asking respondents to name today's date (month, day, year, and season) and the day of the week; visuo-construction (0–1 point) was evaluated by testing the ability to redraw a previously displayed figure; attention (0–5 points) was measured by serial subtraction of 7 from 100 for five times. The total score of cognitive function was the sum of these two dimensions (0–31 points). Higher scores meant better cognitive function.

### 2.3 Exposure assessment

Daily concentrations of ambient particulate matters (PM_1_, PM_2.5_, PM_10_) were estimated at a 0.1° × 0.1° spatial resolution, using aerosol optical depth (AOD; from the Moderate Resolution Imaging Spectroradiometer satellite), meteorological data, land use information, and other spatial and temporal predictors. A detailed description of the estimation and data processing has been published before (Chen et al., 2018a; Chen et al., 2018b; Chen et al., 2018c; Li et al., 2019). Ten-fold cross-validation (CV) was performed to evaluate the predictive accuracy of the method. The results of the 10-fold cross-validation showed that estimate R^2^ and Root Mean Squared Error (RMSE) for monthly PM_1_, PM_2.5,_ and PM_10_ prediction was 71% and 13 μg/m^3^, 86% and 10.7 μg/m^3^, 82% and 19.3 μg/m^3^, respectively, which presented good predictive ability. Based on the longitude and latitude of the city where the participants live, we measured the average of ambient particulate matter (PM_1_, PM_2.5_, PM_10_) concentrations of all the cities. And the exposure windows were 30-day, 60-day, 90-day and 180-day before the interview date.

### 2.4 Covariates

Based on previous studies, we controlled for potential confounding covariates in the analysis. Demographic covariates included age, gender (“Male” and “Female”). Socioeconomic factors included residence (“rural neighborhood” and “urban neighborhood”), marital status (“Married and cohabitating”, and “Divorced, separated, widowed and never married”), education level (“Primary school or below”, “Middle school or above”) and annual household income (categorized by the tertile at the interview year, “Low”, “Median” and “High”). Health behavior variables included smoking status (“Non-smoker” and “Smoker”), drinking status (“Non-drinker” and “Drinker”), body mass index (BMI). Chronic diseases status (“Yes”, “No”) and depressive symptoms status (“Yes”, “No”) were also included in the analysis. Finally, we controlled for regional categories (“East”, “Midland” and “West”) considering the level of economic development with geographical difference.

### 2.5 Statistical analysis

We employed linear mixed models to analyze the impact of PM_1_, PM_2.5,_ and PM_10_ on cognition function. Given the potential regional influence, we set the region as the random effect in the analyses. To investigate the effect of different ambient particulate matters exposure windows, we calculated 30-day, 60-day, 90-day, and 180-day average levels.

Model 1 was developed to incorporate the concentration of ambient particulate matters (PM_1_, PM_2.5_, PM_10_) as the fixed-effect term and set study regions as the random-effect term. In Model 2, we controlled for age, gender, and BMI. Model 3, as a fully adjusted model, additionally adjusted for education level, annual household income, health behavior variables, chronic diseases status, depressive symptoms status, and marital status. All effects were presented as the changes in cognitive function score per interquartile range (IQR) increment in the ambient particulate matters exposures, with corresponding 95% confidence intervals (CIs).

We also performed interaction analyses to assess whether the PM-cognitive function associations were potentially modified by age, gender, residence, education level, annual household income, regional categories, smoking, drinking status, chronic diseases, depressive symptoms, and marital status. We added cross-product terms into separated models to exam the significance of the interaction terms. The interactive analyses were performed using 90-day exposure window of particulate matters as the exposure measure.

We also did sensitivity analyses to examine the reliability of the results. First, we performed the multivariable analyses of the two dimensions of cognitive function, respectively. The model of multivariable analyses adjusted for education level, annual household income, health behavior variables, chronic diseases status, depressive symptoms status, and marital status. A previous study found that depressive symptoms play mediating roles in the effects of air pollution on cognitive function (Tallon et al., 2017). So, we performed another sensitivity analysis to exclude participants with depressive symptoms and examine the effects of ambient particulate matter on the cognitive function of participants without depressive symptoms.

All statistical analyses were completed using R “lme4” and “lmerTest” packages (version 4.0.1).

## 3 Results

**Fig. 1:**
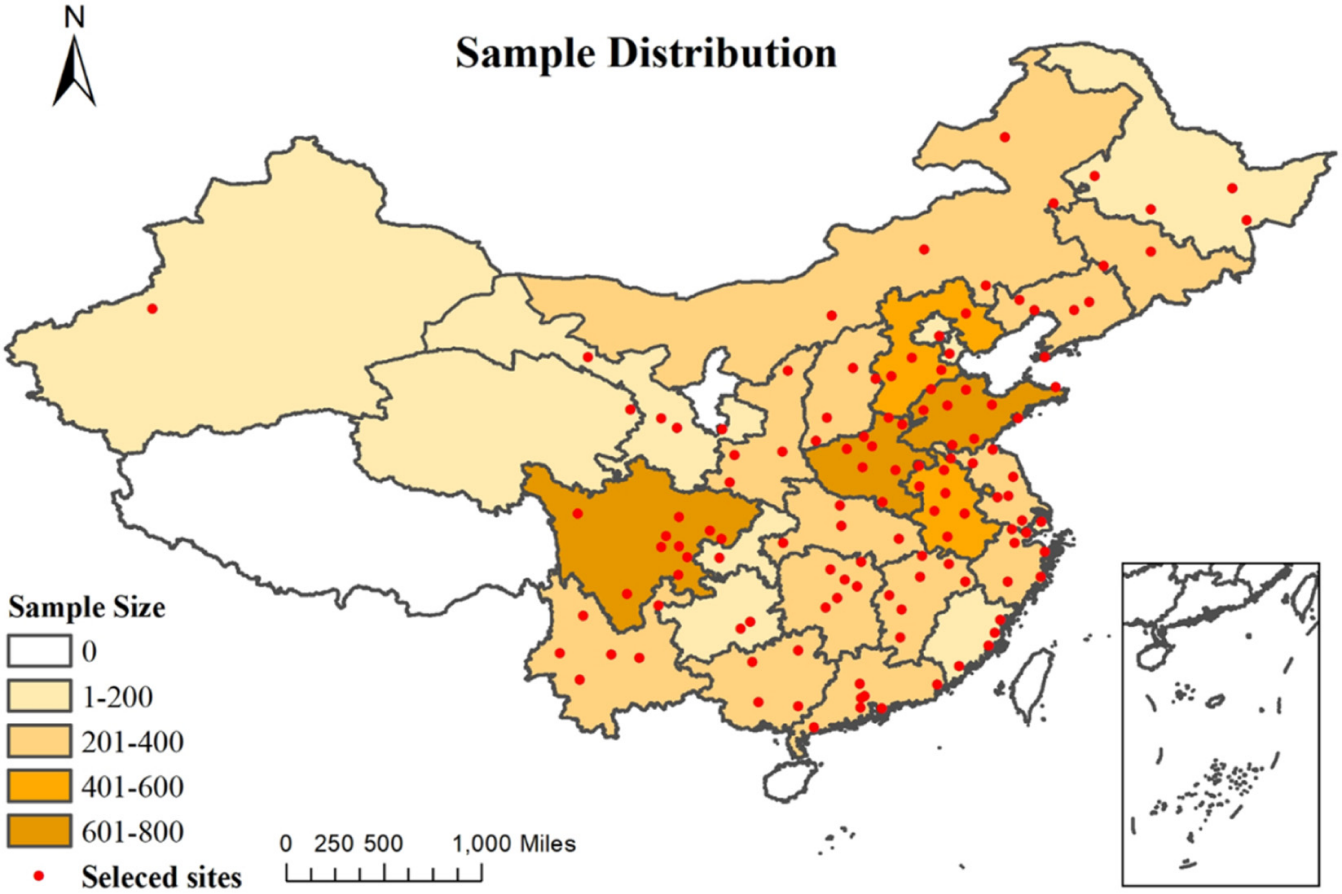
Sample distribution.

There were 7928 participants involved in this study from Wave1 to Wave3 of CHARLS. Fig. 1 showed the distribution of 7928 participants from 28 provinces of China. The basic characteristics of study participants are presented in Table 1. The mean age of participants was 57.60 (SD = 8.46), 4147 (52.31%) of them were male. The majority of participants lived in rural areas (60.91%), and had primary or lower educational status (60.28%). The mean BMI is 23.88 kg/m^2^ (SD = 8.46), 69.49% of participants reported a history of chronic disease, and 32.80% reported a history of depressive symptoms.

**Table 1:** Basic characteristics of study participants.

Characteristics	N	%
Age, mean ± SD, year	57.60 ± 8.46	
<60	4815	60.73
≥60	3113	39.27
Gender		
Male	4147	52.31
Female	3781	47.69
BMI, mean ± SD, kg/m^2^	23.8 ± 3.87	
Residence		
Rural	4829	60.91
Urban	3099	39.09
Marital status		
Married/cohabitating	7200	90.82
Divorced/separated/widowed/never married	728	9.18
Educational level		
Primary school or below	4779	60.28
Middle school or above	3149	39.72
Annual household income		
Low	2648	33.40
Middle	2798	35.29
High	2482	31.31
Smoking status		
Non-smoker	4365	57.58
Smoker	3363	42.42
Drinking status		
Non-drinker	4613	58.19
Drinker	3315	41.81
Region		
Eastern	3187	40.20
Midland	3148	39.71
Western	1593	20.09
Chronic diseases		
No	2419	30.51
Yes	5509	69.49
Depressive symptom status		
No symptoms	5328	67.20
Depressive symptoms	2600	32.80
Total	7928	100

Abbreviations: SD, standard deviation; BMI, body mass index.

Among all participants, 3187 (40.20%) were from eastern China, 3148 (39.71%) from midland, and 1593 (20.09%) from western China. Table S1 showed average of ambient particulate matters concentrations over the previous 30-day to 180-day and cognitive function score. The mean score of participants' cognitive function is 15.54 (SD = 4.86), 15.57 (SD = 5.01), and 14.76 (SD = 5.23) during Wave 1,2 and 3, respectively.

**Fig. 2:**
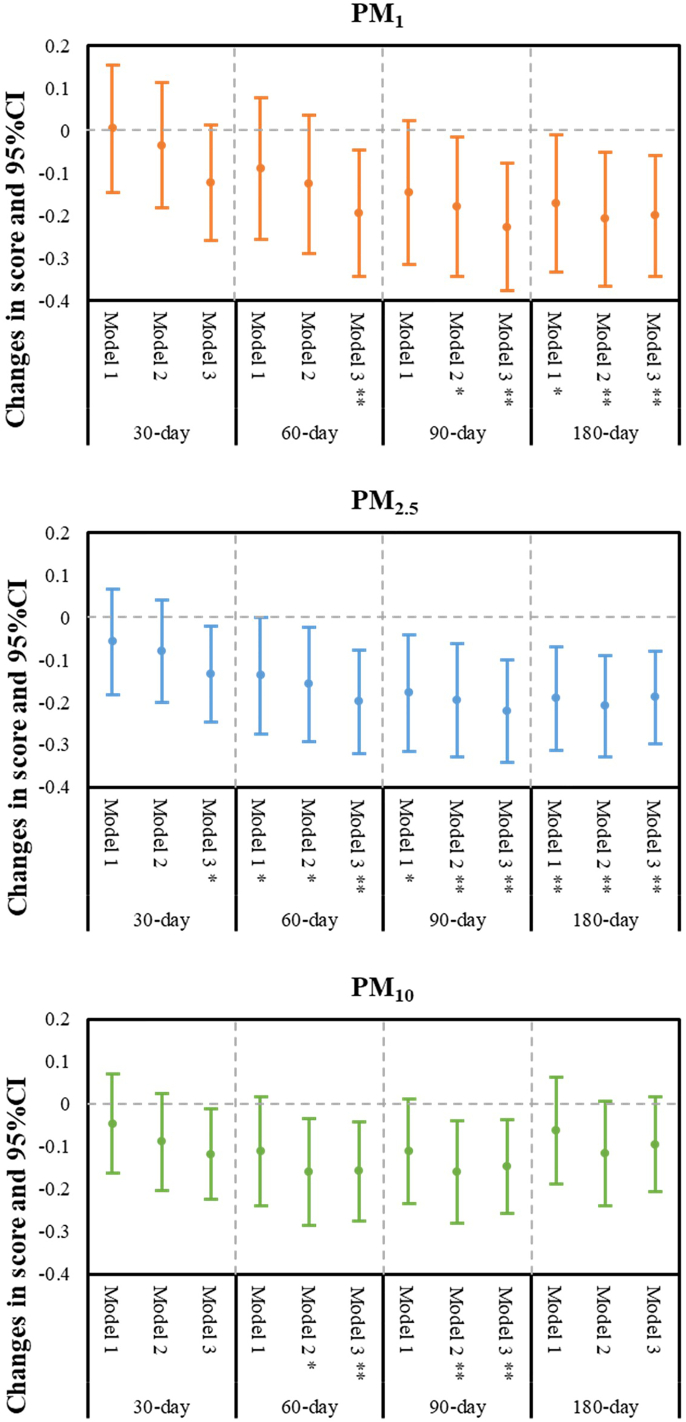
Changes in cognitive function score per IQR increase in ambient particulate matters over preceding period moving averages. Notes: *p < 0.05, **p < 0.01. Abbreviations: CI, confidence interval; IQR, interquartile range; PM_1_, particulate matter with aerodynamic diameter ≤ 1 μm; PM_2.5_, particulate matter with aerodynamic diameter ≤ 2.5 μm; PM_10_, particulate matter with aerodynamic diameter ≤ 10 μm. Model 1 unadjusted; Model 2 adjusted for age, gender, BMI; Model 3 adjusted for age, gender, BMI, residence, marital status, educational level, annual household income, smoking status, drinking status, region, chronic diseases and depressive symptoms status.

The association of different exposure to ambient particulate matters and cognitive function score are presented in Table S3. As for PM_1_, after fully adjusting for covariates, PM_1_ exposure in the 60-day, 90-day, and 180-day windows was significantly associated with a decrease in cognitive function score in model 3, with the greatest effect in the 90-day exposure window (β = −0.227, 95%CI: −0.376, −0.078). For ambient PM_2.5_, the significant exposure windows were 30-day, 60-day, 90-day and 180-day, with the greatest effect in the 90-day exposure window (β = −0.220, 95%CI: −0.341, −0.099) in fully adjusted Model. And for PM_10_, there were similar results in Model 2 and Model 3, an IQR increase in 60-day (Model 3: β = −0.158, 95%CI: −0.274, −0.042) and 90-day (Model 3: β = −0.147, 95%CI: −0.258, −0.037) average was significant associated with a decrease in cognitive function score. In summary, the trend of the ambient particulate matters on cognitive function tends to be similar among these three particulate matters (Fig. 2).

**Fig. 3:**
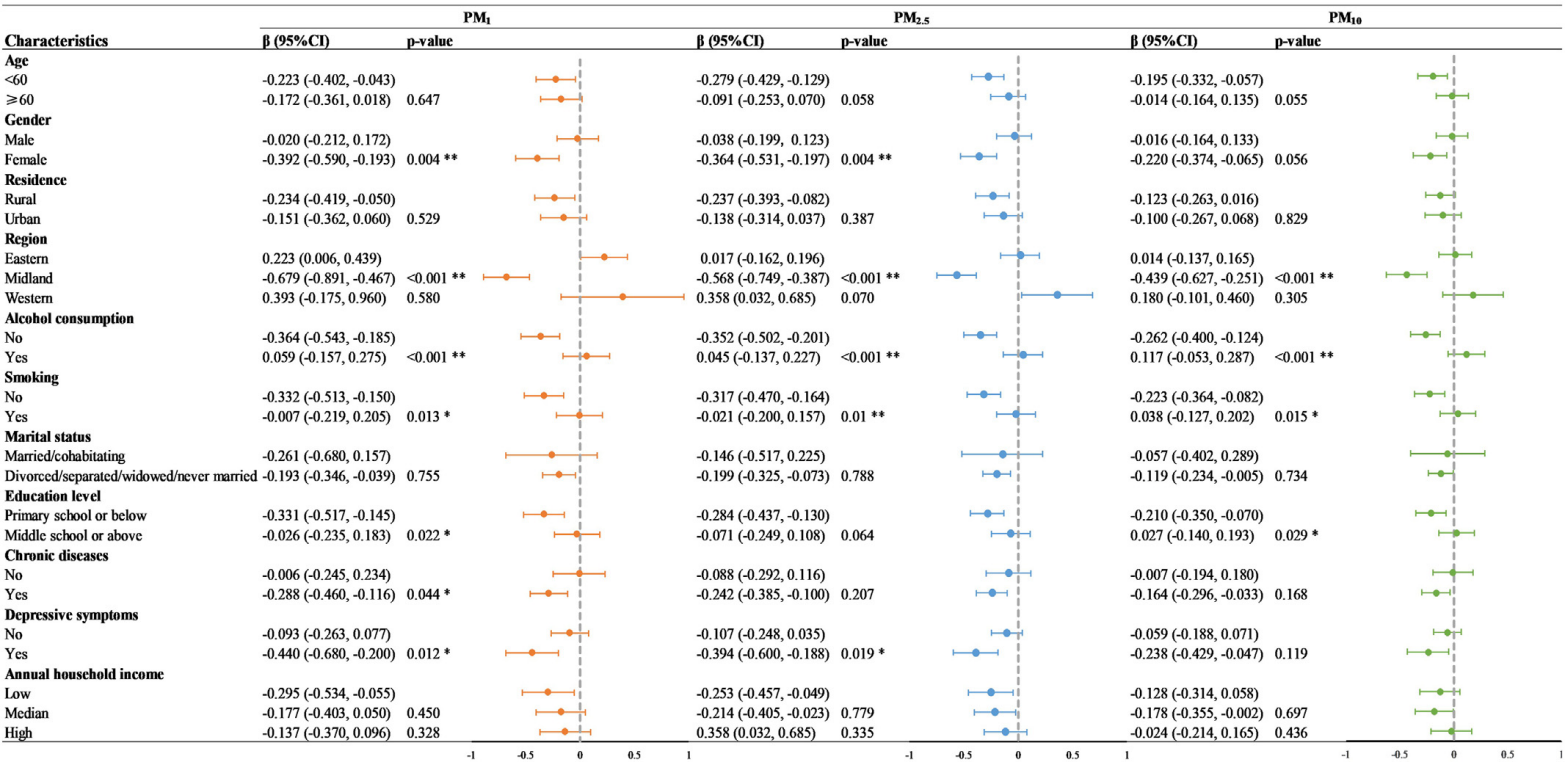
Changes in cognitive function score (95% CI) per IQR increment in 90-day ambient particulate matter levels in multivariate models: modification by participant characteristics. Notes: *p < 0.05, **p < 0.01. Abbreviations: CI, confidence interval; IQR, interquartile range; PM_1_, particulate matter with aerodynamic diameter ≤ 1 μm; PM_2.5_, particulate matter with aerodynamic diameter ≤ 2.5 μm; PM_10_, particulate matter with aerodynamic diameter ≤ 10 μm.

The results of interaction analyses were showed in Fig. 3. Compared to male (β = −0.020, 95%CI: −0.212, −0.172), ambient PM_1_ had a stronger effect on cognitive function decline among female (β = −0.392, 95%CI: −0.590, −0.193). And compared to those from eastern China (β = 0.223, 95%CI: 0.006, 0.439), participants living in the midland of China (β = −0.679, 95%CI: −0.891, −0.467) had significantly larger decreases in cognitive function score with an IQR increase in PM_1_ exposure. We also observed the effect would be stronger among people who had a lower educational level, chronic diseases, depressive symptoms, never smoked or drank. Results were similar to PM_1_, participants who were female, lived in the midland of China, never smoked or drank, or had depressive symptoms were more vulnerable to the impact of ambient PM_2.5_. As for the effect modification results between PM_10_ and cognitive function, compared the participants from eastern China (β = 0.014, 95%CI: −0.137, 0.165), participants from the midland of China (β = −0.439, 95%CI: −0.627, −0.251) showing larger decreases in cognitive function score per IQR increase in PM_10_. And the impact of ambient PM_10_ on the cognitive function decline was greater for those who never smoked or drank or had a lower educational level.

In our sensitivity analyses, the results of the association between an IQR increase in different exposure windows of ambient particulate matters and two dimensions of cognitive function are presented in Fig. S2. And Fig. S3 showed results of the effects of ambient particulate matter on the cognitive function of participants without depressive symptoms. On the episodic memory dimension, 90-day average of PM_1_ (β = −0.139, 95%CI: −0.238, −0.039) and PM_2.5_ (β = −0.142, 95%CI: −0.224, −0.060) had the strongest effect, which was consistent with the results of total cognitive function. On the other dimension, four exposure windows for all three types of particulate matters were associated with mental status decline, and the overall trends were similar to the total cognitive function. As for the results of the association between different exposure windows of ambient particulate matters and cognitive function of participants without depressive symptoms, the trends of all indicators of three types of particulate matters were consistent with the main model analysis. And the most significant negative effect on cognitive function was the 90-day exposure (PM_1_: β = −0.142, 95%CI: −0.320, 0.035; PM_2.5_: β = −0.172, 95%CI: −0.321, −0.023; PM_10_: β = −0.140, 95%CI: −0.279, −0.001;), which was also consistent with main model analysis. The results of sensitivity analyses suggested the robustness of the main analyses results.

## 4 Discussion

To our best knowledge, this was the first study to assess the associations between ambient particulate matters and cognitive function among middle-aged and elderly Chinese. In this longitudinal study, the results showed an adverse effect of ambient particulate matter (PM_1_, PM_2.5_, PM_10_) on cognitive function. After adjusting for potential confounders, the adverse effect of ambient particulate matters on cognitive function became more pronounced.

A growing number of epidemiological studies focused on the effect of exposure to air pollution on cognitive function, however, less studies paid attention to PM_10_, fewer studies focused on PM_1_ (Power et al., 2016). A prospective cohort study from the Monongahela-Youghiogheny Healthy Aging Team (MYHAT) showed that a higher level of ambient PM_2.5_ would lead to a higher risk of incident MCI (Mild Cognitive Impairment) and dementia, each 1 μg/m^3^ increase in PM_2.5_ concentration was related to an HR of 1.669 (95% CI: 1.298, 2.136) for dementia and an HR of 1.746 (95% CI: 1.518, 2.032) for MCI (Sullivan et al., 2021). Another cohort study conducted in London showed a positive association between PM_2.5_ and memory deterioration among old adults, each 1.1 μg/m^3^ increment in PM_2.5_ concentration was associated to 0.03 (β = −0.03, 95%CI: −0.06, 0.002) decrease in standardized memory score (Tonne et al., 2014). Fewer studies evaluated the association between PM_10_ and cognitive function, and the results are different. A study conducted among 789 women from the SALIA cohort reported exposure to PM_10_ had an adverse effect on visuospatial ability (β = −0.14, 95%CI: −0.26, −0.02) (Schikowski et al., 2015), but no adverse effects on general cognitive function, memory, or executive function. Another study group of 399 women aged 68 to 79 years showed no positive association between PM_10_ and cognitive decline (Ranft et al., 2009). Moreover, far fewer studies are available regarding the direct effect of PM_1_ on cognitive function. In fact, smaller particles are more likely to penetrate the body and cause stronger biological effects, such as oxidative stress and internal oxidative inflammatory damage (Valavanidis et al., 2008), which may harm the central nervous system and affect cognitive function (Block and Calderón-Garcidueñas, 2009; Genc et al., 2012). Existing studies confirmed that exposure to PM_1_ were associated with cardiovascular and cerebrovascular diseases (Perez et al., 2009; Perez et al., 2012). And PM_1_ may indirectly affect cognitive function by causing cardiovascular and cerebrovascular diseases. Therefore, it is entirely possible to establish the relationship between PM_1_ and cognitive impairment, but this conclusion needs to be further confirmed by other studies.

The adverse effect of ambient particulate matters on cognitive function can be modified by many factors. In the interaction analyses, we found a significantly larger adverse effect of ambient particulate matters (PM_1_ and PM_2.5_) on cognitive function in female than male. Previous studies showed women were more susceptible to the adverse effect of air pollution (Clougherty, 2010), and this sexual discrepancy may be attributed to physiologic characteristics (e.g., hormonal status, body size) and lifestyle characteristics (e.g., physical activity, smoking and drinking) (Clougherty, 2010). In addition, the results showed that people who drank or smoked were less affected by the effects of ambient particulate matters (PM_1_, PM_2.5_, PM_10_) on cognitive decline than those who never drank or smoked. Existing evidence found that nicotine has positive effects on certain cognitive domains, including working memory and executive function, due to its short-term effects on the cholinergic system and may have neuroprotective effects under certain conditions (Swan and Lessov-Schlaggar, 2007). And another study found low-to-moderate consumption of alcohol was associated with better cognition (Evans and Bienias, 2005).

Furthermore, in the interaction analyses, we found participants in the midland of China may be more vulnerable to ambient particulate matters (PM_1_, PM_2.5_, PM_10_) than those in eastern China. The different level of air pollution in different regions may lead to this result (Azimi et al., 2019). With the rapid development of China's economy, environmental externality is increasingly prominent, and environmental externality is accompanied by environmental inequality (Zhao et al., 2014a). At the beginning of the 20th century, the midland of China played an important role in China's regional economic development. With the acceleration of the modernization of the eastern coastal region and the implementation of China's western development strategy, the economic development of the midland region lagged behind. In recent years, industrialization has developed rapidly in midland of China, but its negative effect is the destruction of the environment and severe air pollution.

Although many studies have explored the relationship between air pollution and cognitive function, the underlying biological mechanisms are not fully understood (Schikowski and Altuğ, 2020). There are different hypotheses about the mechanisms by which air pollution affects cognitive decline. First, ambient particulate matters are small enough to penetrate different tissue chambers in the lungs and eventually enter the systematic circulation and translocate to other organs, including the brain, and ambient particulate matters can also be transferred directly to the brain via the olfactory nerve, where they may be deposited (Elder et al., 2006; Fagundes et al., 2015). Particulate matters can be phagocytosed by macrophages and dendritic cells and activate macrophage inflammatory cytokines, thereby causing neuro-inflammation and oxidative stress (Peters et al., 2006). Oxidative stress and neuro-inflammation induced by these particulates in the brain can harm the central nervous system (CNS) (Block and Calderón-Garcidueñas, 2009; Genc et al., 2012). And damage to the central nervous system can lead to neurodegenerative diseases (Genc et al., 2012). Second, air pollution may indirectly affect cognitive function by causing cardiovascular and cerebrovascular diseases (Hüls et al., 2018; Saito and Ihara, 2016; Toledo et al., 2013). Cognitive degenerative diseases are associated with extracellular amyloid-β protein (Aβ) deposition in blood vessels, as well as intracellular tau deposition in neurofibrillary tangles (Bell and Zlokovic, 2009; Honjo et al., 2012). Air pollution-induced cardiovascular diseases such as hypertension and diabetes are linked to amyloid-β protein (Aβ) deposition, which may lead to brain dysfunction (Genc et al., 2012; Gottesman et al., 2017). Air pollution is considered to be a modifiable cerebrovascular and neurodegenerative risk factor (Béjot et al., 2018). And there is evidence that vascular risk factors and cerebrovascular diseases may accelerate Aβ production, aggregation, and deposition to influence the pathology and symptomatology of Alzheimer's disease (AD) (Honjo et al., 2012; Selkoe, 2000). The effect mechanisms of air pollution on cognitive function need to be clarified by further biological and epidemiological studies in the future.

Despite the innovations and strengths of this study, some limitations also existed. First, although we use satellite-based spatiotemporal models to estimate the PM level, failure to account for the differences in exposure of participants from the same city may decrease the accuracy of results. Second, exposure indicators in this study were matched according to the address of each respondent at the time of the survey, residence change may lead to misclassification. Additionally, although our study has controlled several important confounders, some confounding factors that may influence the results like dietary habit were not considered (Strasser et al., 2016). Finally, the air pollutants only include ambient particulate matter, the effect of other air pollutants on cognitive function can be further studied in the future.

## 5 Conclusion

In general, this study revealed that exposure to ambient particulate matter (PM_1_, PM_2.5_, and PM_10_) for a certain period would significantly decrease cognitive function among middle-aged and elderly Chinese. And the negative effect of particulate matters becomes more pronounced among people who lived in the midland of China, never drank or smoked. The results of this study further supplement existing evidence on the effects of air pollution, especially PM_1_, on cognitive impairment among the middle-aged and elderly in developing countries.

## Funding

This research was supported by the 10.13039/100000865Bill & Melinda Gates Foundation [Grant Number OOP1148464]; Wuhan Center for Disease Control & Prevention [Grant Number 1602-250000196]; Wuhan Municipal Health Commission [Grant Number WY19A01]; Emergency Response Office, Health Commission of Hubei (Health Emergency Response Project of Infectious Disease Risk Investigation and Governance System Construction).

## CRediT authorship contribution statement

Conception and design of study: Hao Xiang, Yifan Yao;

Collating data: Yifan Yao, Kai Wang;

Analysis and/or interpretation of data: Yifan Yao;

Drafting the manuscript: Yifan Yao;

Revising the manuscript critically: Hao Xiang, Kai Wang;

Approval of the version of the manuscript to be published (the names of all authors must be listed): Yifan Yao, Kai Wang, Hao Xiang.

## Declaration of competing interest

All authors declare that there are no conflicts of interest in this study.

## Appendix A Supplementary data

Image 1
