# Supplementary material for: Association between cognitive function and ambient particulate matters in middle-aged and elderly Chinese adults: Evidence from the China Health and Retirement Longitudinal Study (CHARLS)
Source: Sci Total Environ. 2022 Jul 1;828:154297. doi: 10.1016/j.scitotenv.2022.154297 (PMC9112163; doi:10.1016/j.scitotenv.2022.154297)
Supplement: Supplementary file 1 [file mmc1.docx]

**Supplementary Materials**

**Table S1.** Descriptive statistics for ambient particulate matters concentrations over preceding period and cognitive function scores

**Table S2.** Changes in episodic memory and mental status score (95% CIs) per IQR increase in ambient particulate matters over preceding period moving averages after adjusting for age, gender, BMI, residence, marital status, educational level, smoking status, drinking status, region, chronic diseases and depressive symptoms status

**Table S3.** Changes in cognitive function score (95% CIs) per IQR increase in ambient particulate matters over preceding period moving averages

**Fig. S1.** Flowchart of participants’ selection.

**Fig. S2.** Changes in episodic memory and mental status score (95% CIs) per IQR increase in ambient particulate matters over preceding period moving averages after adjusting for age, gender, BMI, residence, marital status, educational level, smoking status, drinking status, region, chronic diseases and depressive symptoms status.

**Fig. S3.** Changes in cognitive function score (95% CIs) per IQR increase in ambient particulate matters over preceding period moving averages after excluding participants having depressive symptoms.

**Supplementary materials**

**Table S1.**

**Descriptive statistics for ambient particulate matters concentrations over preceding period and cognitive function score.**

| **Factors** | **2011 Wave1** | **2013 Wave2** | **2015 Wave3** |
| --- | --- | --- | --- |
| **PM_1_ (μg/m^3^), mean ± SD** | | | |
| 30-day | 33.56±11.59 | 29.93±10.46 | 29.88±10.02 |
| 60-day | 33.67±10.80 | 30.88±10.00 | 30.26±9.81 |
| 90-day | 34.18±10.22 | 32.05±9.69 | 31.39±9.72 |
| 180-day | 41.29±12.26 | 40.90±12.74 | 39.90±11.83 |
| **PM_2.5_ (μg/m^3^), mean ± SD** | | | |
| 30-day | 44.86±13.98 | 39.28±12.47 | 39.39±11.78 |
| 60-day | 45.34±12.86 | 40.90±11.70 | 40.27±11.38 |
| 90-day | 45.95±11.85 | 42.63±11.24 | 41.85±10.99 |
| 180-day | 54.61±14.07 | 54.77±15.45 | 52.85±13.49 |
| **PM_10_ (μg/m^3^), mean ± SD** | | | |
| 30-day | 101.28±26.07 | 71.32±21.39 | 74.57±23.97 |
| 60-day | 95.22±22.29 | 77.58±22.80 | 79.38±23.51 |
| 90-day | 95.81±22.48 | 83.50±23.98 | 84.28±23.20 |
| 180-day | 101.28±26.07 | 100.07±27.33 | 98.64±25.35 |
| **Cognitive function, mean ± SD** | | | |
| Total score | 15.54±4.86 | 15.57±5.01 | 14.76±5.23 |
| Memory | 7.56±3.26 | 7.72±3.31 | 7.11±3.50 |
| Mental status | 7.98±2.70 | 7.85±2.76 | 7.65±2.79 |

Abbreviations: SD, standard deviation; PM_1_, particulate matter with aerodynamic diameter ≤1 μm; PM_2.5_, particulate matter with aerodynamic diameter ≤2.5 μm; PM_10_, particulate matter with aerodynamic diameter ≤10 μm.

**Table S2.**

**Changes in episodic memory and mental status score (95% CIs) per IQR increase in ambient particulate matters over preceding period moving averages after adjusting for age, gender, BMI, residence, marital status, educational level, smoking status, drinking status, region, chronic diseases and depressive symptoms status.**

|  | **Episodic memory** | | **Mental status** | |
| --- | --- | --- | --- | --- |
| **Pollutants** | **Exposure windows** | **β (95%CI)** | **Exposure windows** | **β (95%CI)** |
|  | 30-day | -0.087 (-0.179, 0.005) | 30-day | -0.034 (-0.096, 0.028) |
| **PM_1_** | 60-day * | -0.125 (-0.224, -0.026) | 60-day | -0.060 (-0.128, 0.009) |
|  | 90-day ** | -0.139 (-0.238, -0.039) | 90-day * | -0.069 (-0.136, -0.002) |
|  | 180-day * | -0.112 (-0.209, -0.015) | 180-day ** | -0.080 (-0.140, -0.020) |
|  |  |  |  |  |
|  | 30-day * | -0.096 (-0.173, -0.019) | 30-day | -0.027 (-0.102, 0.047) |
| **PM_2.5_** | 60-day ** | -0.131 (-0.214, -0.047) | 60-day | -0.059 (-0.140, 0.023) |
|  | 90-day ** | -0.142 (-0.224, -0.060) | 90-day | -0.078 (-0.160, 0.003) |
|  | 180-day ** | -0.118 (-0.192, -0.043) | 180-day * | -0.093 (-0.172, -0.014) |
|  |  |  |  |  |
|  | 30-day | -0.057 (-0.131, 0.017) | 30-day | -0.057 (-0.116, 0.002) |
| **PM_10_** | 60-day | -0.058 (-0.137, 0.021) | 60-day ** | -0.085 (-0.149, -0.021) |
|  | 90-day | -0.037 (-0.112, 0.037) | 90-day ** | -0.092 (-0.153, -0.031) |
|  | 180-day | 0.009 (-0.066, 0.084) | 180-day ** | -0.101 (-0.163, -0.040) |

Notes: *p <0.05, **p <0.01. Abbreviations: CI, confidence interval; IQR, interquartile range; BMI, body mass index; PM_1_, particulate matter with aerodynamic diameter ≤1 μm; PM_2.5_, particulate matter with aerodynamic diameter ≤2.5 μm; PM_10_, particulate matter with aerodynamic diameter ≤10 μm.

**Table S3.**

**Changes in cognitive function score (95% CIs) per IQR increase in ambient particulate matters over preceding period moving averages.**

| **Pollutants** | **Exposure** | **Cognitive function score (range:0-30)** | | |
| --- | --- | --- | --- | --- |
|  | **windows** | **Model 1** | **Model 2** | **Model 3** |
|  | 30-day | 0.006 (-0.145, 0.156) | -0.035 (-0.182, 0.112) | -0.123 (-0.258, 0.012) |
| **PM_1_** | 60-day | -0.088 (-0.255, 0.079) | -0.126 (-0.289, 0.036) | -0.195 (-0.343, -0.047) ** |
|  | 90-day | -0.145 (-0.313, 0.024) | -0.180 (-0.344, -0.016) * | -0.227 (-0.376, -0.078) ** |
|  | 180-day | -0.171 (-0.332, -0.010) * | -0.209 (-0.366, -0.052) ** | -0.201 (-0.344, -0.058) ** |
|  |  |  |  |  |
|  | 30-day | -0.056 (-0.180, 0.067) | -0.080 (-0.201, 0.041) | -0.134 (-0.247, -0.022) * |
| **PM_2.5_** | 60-day | -0.136 (-0.274, 0.002) * | -0.158 (-0.293, -0.023) * | -0.199 (-0.322, -0.076) ** |
|  | 90-day | -0.177 (-0.314, -0.040) * | -0.195 (-0.328, -0.061) ** | -0.220 (-0.341, -0.099) ** |
|  | 180-day | -0.190 (-0.312, -0.068) ** | -0.209 (-0.328, -0.090) ** | -0.188 (-0.297, -0.079) ** |
|  |  |  |  |  |
|  | 30-day | -0.045 (-0.162, 0.072) | -0.089 (-0.203, 0.026) | -0.118 (-0.224, -0.011) |
| **PM_10_** | 60-day | -0.109 (-0.237, 0.019) | -0.159 (-0.285, -0.033) * | -0.158 (-0.274, -0.042) ** |
|  | 90-day | -0.110 (-0.233, 0.013) | -0.160 (-0.280, -0.039) ** | -0.147 (-0.258, -0.037) ** |
|  | 180-day | -0.060 (-0.187, 0.066) | -0.115 (-0.239, 0.008) | -0.095 (-0.207, 0.017) |

Notes: *p <0.05, **p <0.01. Abbreviations: CI, confidence interval; IQR, interquartile range; PM_1_, particulate matter with aerodynamic diameter ≤1 μm; PM_2.5_, particulate matter with aerodynamic diameter ≤2.5 μm; PM_10_, particulate matter with aerodynamic diameter ≤10 μm. Model 1 unadjusted; Model 2 adjusted for age, gender, BMI; Model 3 adjusted for age, gender, BMI, residence, marital status, educational level, annual household income, smoking status, drinking status, region, chronic diseases and depressive symptoms status.


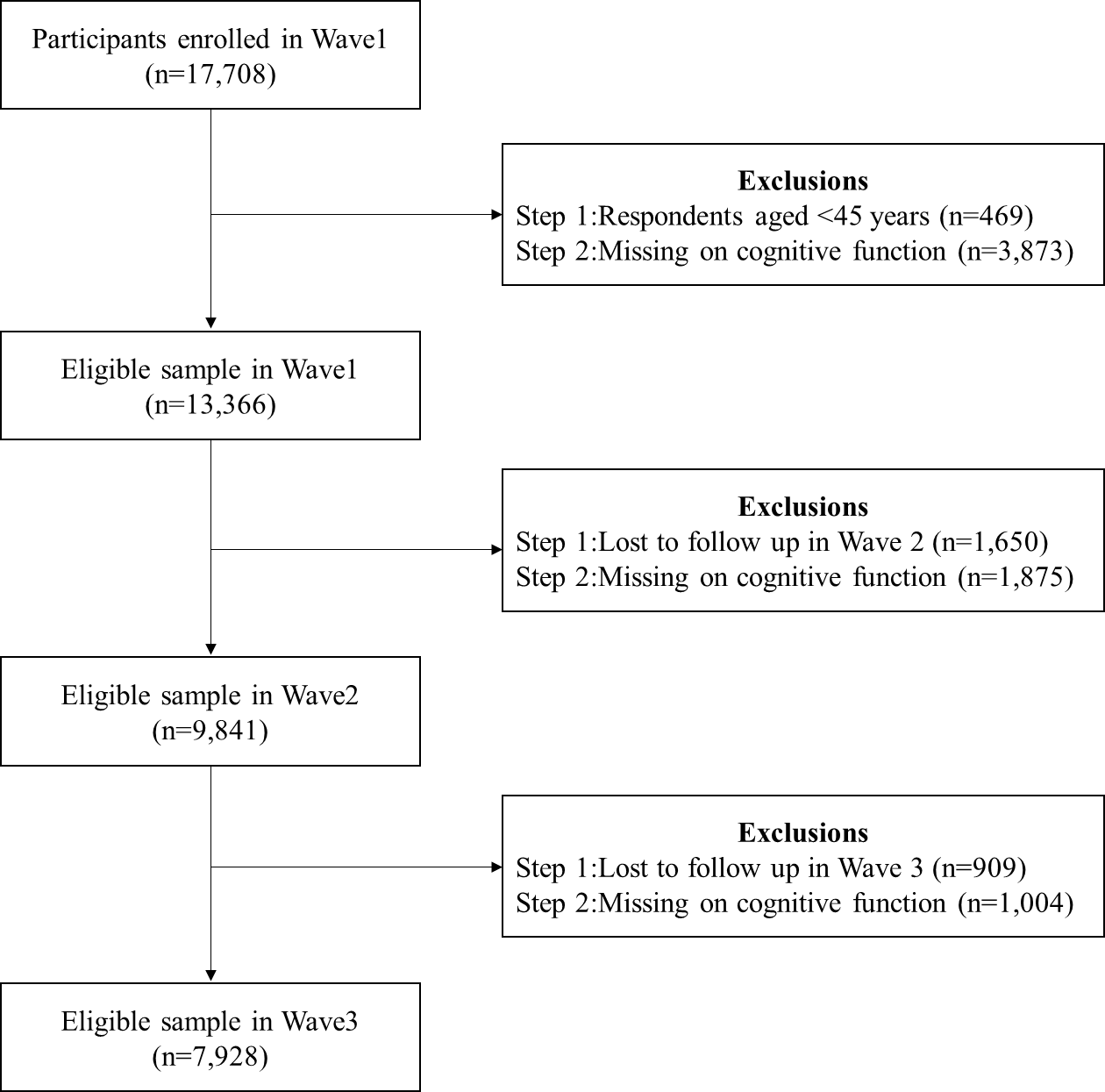


**Fig. S1.** **Flowchart of participants’ selection.**


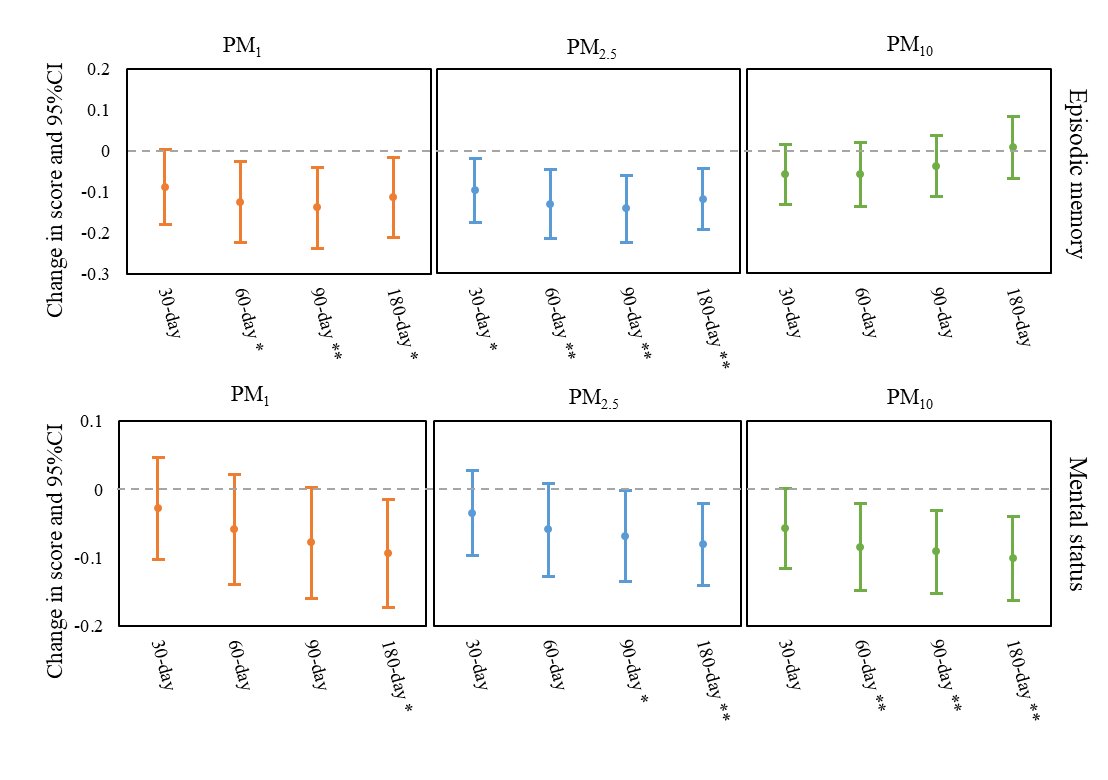


**Fig. S2.** Changes in episodic memory and mental status score (95% CIs) per IQR increase in ambient particulate matters over preceding period moving averages after adjusting for age, gender, BMI, residence, marital status, educational level, smoking status, drinking status, region, chronic diseases and depressive symptoms status.

Notes: *p <0.05, **p <0.01. Abbreviations: BMI, body mass index; CI, confidence interval; IQR, interquartile range; PM_1_, particulate matter with aerodynamic diameter ≤1 μm; PM_2.5_, particulate matter with aerodynamic diameter ≤2.5 μm; PM_10_, particulate matter with aerodynamic diameter ≤10 μm.

**Fig. S3.** Changes in cognitive function score (95% CIs) per IQR increase in ambient particulate matters over preceding period moving averages after excluding participants having depressive symptoms.

Notes: *p <0.05, **p <0.01. Abbreviations: CI, confidence interval; IQR, interquartile range; PM_1_, particulate matter with aerodynamic diameter ≤1 μm; PM_2.5_, particulate matter with aerodynamic diameter ≤2.5 μm; PM_10_, particulate matter with aerodynamic diameter ≤10 μm.
